# Whether the Support Region of Three-Bit Uniform Quantizer Has a Strong Impact on Post-Training Quantization for MNIST Dataset?

**DOI:** 10.3390/e23121699

**Published:** 2021-12-20

**Authors:** Jelena Nikolić, Zoran Perić, Danijela Aleksić, Stefan Tomić, Aleksandra Jovanović

**Affiliations:** 1 Faculty of Electronic Engineering, University of Nis, Aleksandra Medvedeva 14, 18000 Nis, Serbia; zoran.peric@elfak.ni.ac.rs (Z.P.); aleksandra.jovanovic@elfak.ni.ac.rs (A.J.); 2 Department of Mobile Network Nis, Telekom Srbija, Vozdova 11, 18000 Nis, Serbia; danijelaal@telekom.rs; 3 School of Engineering and Technology, Al Dar University College, Dubai 35529, United Arab Emirates; stefan@aldar.ac.ae

**Keywords:** image classification, Laplacian source, neural network, uniform quantization

## Abstract

Driven by the need for the compression of weights in neural networks (NNs), which is especially beneficial for edge devices with a constrained resource, and by the need to utilize the simplest possible quantization model, in this paper, we study the performance of three-bit post-training uniform quantization. The goal is to put various choices of the key parameter of the quantizer in question (support region threshold) in one place and provide a detailed overview of this choice’s impact on the performance of post-training quantization for the MNIST dataset. Specifically, we analyze whether it is possible to preserve the accuracy of the two NN models (MLP and CNN) to a great extent with the very simple three-bit uniform quantizer, regardless of the choice of the key parameter. Moreover, our goal is to answer the question of whether it is of the utmost importance in post-training three-bit uniform quantization, as it is in quantization, to determine the optimal support region threshold value of the quantizer to achieve some predefined accuracy of the quantized neural network (QNN). The results show that the choice of the support region threshold value of the three-bit uniform quantizer does not have such a strong impact on the accuracy of the QNNs, which is not the case with two-bit uniform post-training quantization, when applied in MLP for the same classification task. Accordingly, one can anticipate that due to this special property, the post-training quantization model in question can be greatly exploited.

## 1. Introduction

Neural networks (NNs) have achieved remarkable success in a wide range of real-world applications. However, their application might be limited or impeded in edge devices with a constrained resource, such as IoT and mobile devices [1,2,3,4,5,6]. Although on such resource-constrained devices, decreased storage and/or computational costs for NNs are indispensable, the accuracy of NN can be severely degraded if the pathway toward this decrease is not chosen prudently [2,4,6]. The rapid proliferation of IoTs, as predicted by [7], additionally highlights the increasing importance of the edge infrastructure and a needful migration of NNs to the very end devices. A training of NNs on the edge devices presents the very idea of edge computing, which is, for the edge devices, competitive to the cloud computing from the aspects of latency, memory footprint and power consumption [1,3,5,6]. In artificial intelligence (AI) algorithms that are running in the cloud, data have to be sent over the Internet to the cloud, causing latency and thus preventing AI-based real-time applications, as well as security problems. The main advantage of edge computing lies in agile, preferably not belated interactions with end devices [1,3]. Edge computing and cloud computing are not mutually exclusive. On the contrary, edge computing extends the cloud, making it as close as possible to heterogeneous end devices or end users [1,3].

An efficient edge computing with highly accurate NNs requires embarking on a comprehensive rethinking of the NN design and adopting different compression techniques, such as pruning, knowledge distillation and quantization [2,8,9,10,11,12,13,14,15,16,17,18,19,20,21]. Relying on an abundance of the previous conclusions about quantization for traditional network solutions [22,23,24,25,26,27,28,29,30,31,32], further improvements in the field of NNs, especially in NNs intended for edge devices, can be intuitively driven by the prudent application of post-training quantization. Post-training quantization is especially convenient as there is no need for retraining NN, while the memory size required for storing the weights of the quantized neural network (QNN) model can be significantly reduced compared to the baseline NN model utilizing 32-bit floating-point (FP32) format [6,14,15,19,33].

Numerous recent papers have already confirmed ample opportunities for the NN parameters compression by means of post-training quantization, moving from single-precision FP32 to lower-bit presentations, thereby notably reducing the memory costs while degrading the NN accuracy to some extent [6,14]. In other words, various quantization methods result in different QNN performances, where, with extremely low-bit quantization models, it is hard to achieve accuracy comparable with that of FP32 models [2,6]. Namely, an important challenge in post-training quantization is that it can lead to significant performance degradation, especially in ultra-low precision settings. To cope with this, inspired by the conclusions from classical quantization, numerous papers have addressed the problem of minimizing the inevitable post-training quantization error (see, for instance, [6,12,15,33]).

The amount of quantization error is directly conditioned by the quantizer parameterization and the source of the data being quantized [31]. By determining signal to quantization noise ratio (SQNR), which formally quantifies how well original data are quantized, one can improve the quantizer performances by changing its parameters in a way to maximize SQNR, or equally, to minimize the distortion. Recently, it has been shown in [34] that the performance of the quantizer alone might not necessarily affect the accuracy degradation the most. In fact, a lot of the QNN models from the literature, e.g., from [10,11,12,13,14,15,16,17,18,19,20], primarily targeted an acceptable or high accuracy of QNN, that is, an accuracy comparable to one of the initial FP32 NN models. Since the original parameters of NN are typically stored in FP32 format, we believe that there is a large potential opportunity for their compression, as long as the quantized parameters of QNN are close to the original ones. This becomes important when it comes to the large parameter sets of the NN model, which are preferable to be processed as fast as possible. Hence, low-bit quantization can be considered as an attractive and powerful technique that provides such a compressed NN, i.e., QNN, to fit in the edge device with as little loss of performance/accuracy as possible. However, there is still a large gap between the performance of QNNs and full-precision NNs. To bridge this gap, extensive research in this field should be performed.

The rest of the paper is structured as follows: First, related previous work is summarized in Section 2, along with our concrete motivation. Then, in Section 3, the design of the symmetrical three-bit UQ is described, and the closed-form formulas for calculating its theoretical performance for the assumed Laplacian pdf are derived. In Section 4, a concise description of the two utilized NN architectures, as well as the MNIST and Fashion-MNIST datasets, are provided. The procedure of post-training three-bit uniform quantization of weights is described in Section 5, where, to be as specific as possible, the pseudocode is also provided. The numerical results of the paper are presented and discussed in Section 6. Finally, in Section 7, the paper’s goals are summarized, conclusions on our research results are derived, and directions for future work are provided as well.

## 2. Related Work and Motivation

In traditional low-bit quantization, a very small number of bits per sample are used to represent the data being quantized (less than or equal to 3 bit/sample) [31]. Since the NN compression by means of post-training quantization is gaining momentum, we believe that substantial progress might be accomplished by the proper utilization of the simple uniform quantizer model. For that reason, we are interested in analyzing whether such a simple three-bit uniform quantizer model can preserve the accuracy of NNs to a great extent, regardless of the choice of its key parameter (support region threshold).

The uniform fixed-rate scalar quantizer, or briefly, uniform quantizer (UQ), is the simplest quantizer model, which, for a given bit rate, *R* is characterized with only one parameter—the support region threshold. However, its design, or determining the support region threshold to provide the smallest possible mean-squared error (MSE) distortion, is not simple for source densities with infinite support [22,25,35,36]. The reason is that expressions for the MSE distortion of UQ for source densities with infinite support are not simple, so the distortion minimization per support region threshold does not generally provide the derivation of a closed-form expression for the optimal support region threshold [22,35,36]. Note that the above-mentioned problem is more prominent as *R* increases since the expression for the UQ performance assessment obtains more terms, and, accordingly, determining the optimal support region threshold becomes more complex. As highlighted in [34,35], this issue has greater importance in uniform quantization than in a nonuniform one. However, the question that arises in this paper is whether it is of the utmost importance to determine the optimal support region threshold value to achieve some predefined performance of post-training three-bit uniform quantization. To the best of the authors’ knowledge, the analysis of the post-training performance from the perspective of choosing the support region has not been reported thus far for the three-bit uniform quantization. It is worth highlighting here that in [34], we have analyzed whether it is possible to apply the simplest uniform quantization with the bit rate of *R* = 2 bit/sample for representing the weights of the trained multi-layer perceptron (MLP) and to significantly preserve the accuracy. Since, in [34], we showed that the choice of the support region has a large effect on the QNN accuracy, in this paper, still focusing on low-bit weight presentations, our goal is to determine whether completely novel insights and conclusions can be derived in the case where three-bit uniform quantization is utilized for the same classification task.

In [34], we showed that with the two-bit uniform quantizer adjusted for the Laplacian distribution and utilized in NN post-training, the accuracy of the considered three-layer fully connected (FC) QNN model is very dependent on the proper choice and the definition of the support region, as well as on the step size of the quantizer in question. This has inspired our further research on three-bit uniform quantization with the doubled number of representation levels. More precisely, with the two-bit UQ used for the compression of weights from the MLP model, we have managed to significantly preserve the accuracy, which is degraded by slightly more than 1%, while the weights are compressed 16 times relative to the FP32 format. However, we showed in [34] that an unsuitable choice of the support region could significantly degrade the accuracy (more than 3.5%), that is, the performance of the post-training two-bit uniform quantization for the assumed MLP model trained on MNIST. Therefore, to keep the quantized weights as close as possible to the original ones and target at least half-reduced accuracy degradation from that given in [34], in this paper, we examine whether this goal is achievable with a far simpler and less stringent choice of the support region threshold value of the three-bit UQ. Since SQNR is a key quantization indicator, of particular interest is to show how the selection of the key parameter affects both the SQNR and the accuracy of QNN.

As one can anticipate, assigning one additional bit for weights quantization is important for all layers, starting from the first layer (crucial to the following layers since it needs to represent the initial weights as well as possible) to the last layer that directly computes the final outputs. Let us highlight that in [34], we derived one interesting conclusion about the layer-wise adaptation since we noticed that the distributions of weights varied across different layers of the MLP. In particular, we have noticed that at the third layer of the MLP, the greatest deviation from the Laplacian distribution exists and that distribution approaches the uniform-like one, which is therefore convenient for the application of uniform quantization addressed in this paper.

Besides improvement through a thoughtful quantizer reparameterization, as an added convenience for NN accuracy preservation, one can anticipate NN reparameterization by means of NN layer feature scaling or the so-called normalization of NN, which is utilized in this paper. Namely, NN normalization methods have already been confirmed to work well empirically [37], whereby a lot of them implicitly assume that the distributions of normalized NN parameters, primarily weights, initially have zero mean and unit variance. Keeping in mind that the weights’ distribution can closely fit some well-known probability density functions, such as the Laplacian probability density function (pdf) is [2], in this paper, as in [12,13,34,38,39,40], we assume the Laplacian-like distribution for experimental weights’ distribution and the Laplacian pdf for the theoretical distribution of weights to estimate the performance of the three-bit uniform quantizer (three-bit UQ) in question. Our motivation to address the simplest UQ also stems from the fact that UQs are not optimal for nonlinear distributions. Hence, our goal is to investigate the viability of the accuracy of the QNN in the case of uniformly quantized weights from nonlinear distributions, such as the Laplacian one. Moreover, to further widen our numerical result analysis compared to the one from [34], in this paper, we analyze the performance of post-training three-bit uniform quantization for the convolutional neural network (CNN) applied to the MNIST dataset classification problem. More details on utilized MLP and CNN are provided in Section 4.

## 3. Design of Symmetric Three-Bit Uniform Quantizer for the Purpose of NN Weights Compression

For symmetrical data distributions, symmetrical quantizers with an even number of quantization steps are preferable [14,24,25,26,27,28,29,32,34,35,38,40]. Since it can be expected that most of the real data is asymmetrical, one could not conjecture that the preferable quantizer is also asymmetrical. For a better understanding of the quantization process, which accounts for the real but not necessarily symmetric data, in this section, we describe the symmetric three-bit uniform quantizer model that is designed for the Laplacian pdf. In the later sections, we describe its adjustment to the real data, i.e., to the weights of the pretrained NN. Since low-bit quantization can entail substantially diminishing in terms of SQNR, we describe the theoretical and experimental scenarios that target three-bit uniform quantization of pretrained FP32 weights of two NNs (MLP and CNN). As already mentioned, the weight distribution can closely fit some well-known pdfs, such as Laplacian pdf is [2]. Accordingly, as in [12,13,34,38,39,40], we assume the Laplacian-like distribution for the experimental distribution of weights and the Laplacian pdf for the theoretical distribution of weights to estimate the performance of our three-bit UQ in question.

**Figure 1:**
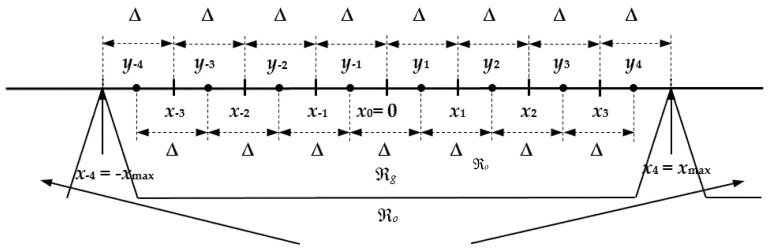
Granular and overload regions of the symmetric uniform quantizer for *R* = 3 bit/sample and partition into cells of equal widths.

Firstly, we invoke the well-known preliminaries about uniform quantization and an *N*-level symmetrical uniform quantizer $Q_{N}^{\mathrm{UQ}}$. During the quantization procedure, an input signal amplitude range is divided into a granular region ℜ*_g_* and an overload region ℜ*_o_*, separated by the support region threshold *x*_max_ [31]. For a given bit rate of *R* = 3 bit/sample, with our symmetrical three-bit UQ, ℜ*_g_* is partitioned into *N* = 2*^R^* = 8, equally spaced, nonoverlapped and bounded in width granular cells (see Figure 1). The *i*^th^ granular cell of the three-bit UQ is defined as:

(1) $${ℜ}_{i}=\{x\;|x\;\in \;[-x_{\max},x_{\max}],Q_{N}^{\mathrm{UQ}}\left(x\right)=y_{i}=\left(2i-1\right)x_{\max}/8\},{ℜ}_{i}\cap {ℜ}_{j}=\emptyset ,\;i\neq j$$

while ${\left\{{ℜ}_{i}\right\}}_{i=-N/2}^{-1}$ and ${\left\{{ℜ}_{i}\right\}}_{i=1}^{N/2}$ denote the granular cells in the negative and positive in amplitude regions, which are symmetrical. This symmetry also holds for the overload quantization cells, that is, for a pair of cells with unlimited widths in the overload region, ℜ*_o_*, defined as:

(2) $${ℜ}_{0}=\{x|x\;\notin [-x_{\max},x_{\max}],Q_{N}^{\mathrm{UQ}}\left(x\right)=y_{N/2}=7/8\;x_{\max},\;x>0\;\vee Q_{N}^{\mathrm{UQ}}\left(x\right)=y_{-N/2},\;x<0\}$$

The UQ model implies that the granular cells have equal widths (Δ), the output levels are the midpoints of the corresponding granular cells, and the code book $Y\equiv \left\{y_{-N/2},\ldots y_{-1},y_{1},\ldots ,y_{N/2}\right\}\subset \mathbb{R}$ contains *N* representation levels, denoted by *y_i_*. In other words, by recalling the quantization procedure, the parameters of the symmetrical three-bit UQ can be calculated from:

(*a*) Δ*_i_* = Δ = *x*_max_/4, *i* ∊ {1, 2, 3, 4},
(*b*) −*x_−i_* = *x_i_*, *x_i_* = *i·*Δ = *i·x*_max_/4, *i* ∊ {0, 1, 2, 3, 4},
(*c*) −*y_−i_* = *y_i_*, *y_i_* = (*x_i_* + *x_i_*_−1_)/2 = (2*i* − 1)·*x*_max_/8, *i* ∊ {1, 2, 3, 4}.

The most common reason why it is convenient to deal with the zero-mean symmetric quantizer is that the codebook and the quantizer main parameter set can be halved since only the positive or the absolute values of the parameters should be stored. Recalling that *x*_max_ = *x*_4_ denotes the support region threshold of the three-bit UQ, one can conclude from rules (*a*)–(*c*) that *x*_max_ completely determines the cell width Δ, the cell borders *x_i_*, *i* ∊ {0, 1, 2, 3, 4} and the representation levels (output levels) *y_i_*, *i* ∊ {1, 2, 3, 4} of the three-bit UQ (see Figure 1). Accordingly, as we have already mentioned, *x*_max_ is the key parameter of the uniform quantizer.

**Figure 2:**
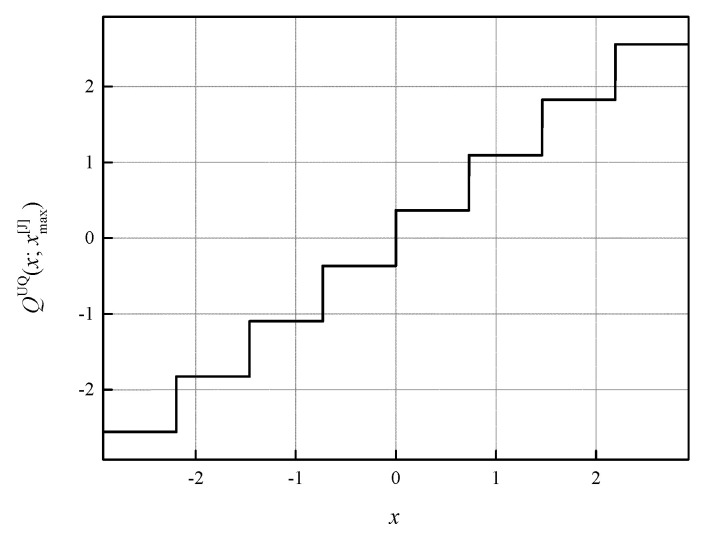
Transfer characteristic of the uniform quantizer *Q*^UQ^(*x*; *x*_max_[J]) for *R* = 3 bit/sample and for [−*x*_max_[J], *x*_max_[J]] = [−2.9236, 2.9236].

For the given *x*_max_, UQ is also well described by its transfer characteristic. The transfer characteristic of our symmetric three-bit UQ is given by:

(3) $$Q^{\mathrm{UQ}}\left(x;\;x_{\max}\right)=\left\{ \begin{array}{l} \left(\left\lfloor \frac{4\left|x\right|}{x_{\max}}\right\rfloor +\frac{1}{2}\right)\frac{x_{\max}}{4}\;\mathrm{sgn}(x),\;\;\left|x\right|\leq x_{\max}\\ \;\frac{7}{8}x_{\max}\;\mathrm{sgn}(x),\;\;\left|x\right|>x_{\max} \end{array}\right.$$

where notation *N*, indicating the number of quantization levels, is omitted due to the simplicity reasons. For *x*_max_ = *x*_max_[J], it is illustrated on Figure 2 and is denoted by *Q*^UQ^(*x*; *x*_max_[J]), where the notation [J] comes from the name of the author of [31]).

An important aspect of our interest in the three-bit UQ design that dictates assumptions about parameters of the quantizer in question has emerged from the maximal SQNR or the minimal overall distortion, that is, in the case where *x*_max_ has an optimal value. For a given bit rate of *R* = 3 bit/sample, the MSE distortion of the three-bit UQ for a source density with infinite support can be expressed as a sum of the granular and the overload distortion from the granular and overload regions, respectively [22,24,31,35,36]. To determine the total distortion of our three-bit UQ that is a sum of the granular and the overload distortion, denoted, respectively, by *D*_g_^UQ^ and *D*_o_^UQ^, we begin with the basic definition of the distortion, given by [31]:

(4) $$D_{g}^{\mathrm{UQ}}=2\sum_{i=1}^{4}\int_{x_{i-1}}^{x_{i}}{\left(x-y_{i}\right)}^{2}p\left(x\right)dx$$

(5) $$D_{o}^{\mathrm{UQ}}=2\int_{x_{4}}^{\infty }{\left(x-y_{4}\right)}^{2}p\left(x\right)dx$$

Let us recall that the cell borders and representation levels are functions of *x*_max_. Accordingly, an inspection of Formulas (4) and (5) reveals that *x*_max_ appears not only in the limits of integrals but also in integrands. Hereof, despite the simplicity of the UQ model, determining *x*_max_ analytically to provide the minimal MSE distortion is still difficult or even impossible. The value of *x*_max_ determined by Jayant [31] is a result of numerical distortion optimization. Hui had analytically obtained the equation for *x*_max_ of the symmetrical *N*-level asymptotically optimal UQ that is designed for high bit rates and an input signal with the Laplacian pdf of zero mean and unit variance [22], which, for *N* = 2*^R^* = 8, gives:

(6) $$x_{\max}{}^{\left[H\right]}=\sqrt{2}\ln\left(8\right)$$

As we pointed out earlier, we assume the unrestricted Laplacian pdf of zero mean and variance *σ*^2^ = 1 for the input data distribution in the theoretical analysis of our three-bit UQ:

(7) $$p(x)=\frac{1}{\sqrt{2}\sigma }\exp\left\{-\frac{\sqrt{2}x}{\sigma }\right\}$$

First, to simplify our derivation, let us define that it holds that *x*_5_ = ∞. In other words, *x*_5_ denotes the upper limit of the integral in Equation (5). By introducing *x*_5_, the total distortion of our symmetrical three-bit UQ can be rewritten as:

(8) $$D^{\mathrm{UQ}}=2\sum_{i=1}^{5}\int_{x_{i-1}}^{x_{i}}x^{2}p\left(x\right)dx-4\left(\sum_{i=1}^{4}y_{i}\int_{x_{i-1}}^{x_{i}}xp\left(x\right)dx+y_{4}\int_{x_{4}}^{\infty }xp\left(x\right)dx\right)+2\left(\sum_{i=1}^{4}y_{i}{}^{2}\int_{x_{i-1}}^{x_{i}}p\left(x\right)dx+y_{4}{}^{2}\int_{x_{4}}^{\infty }p\left(x\right)dx\right)$$

or shortly as:

(9) $$D^{\mathrm{UQ}}={ϒ}^{I}-4\left(\sum_{i=1}^{4}y_{i}{ϒ}_{i}^{\mathrm{II}}+y_{4}{ϒ}_{5}^{\mathrm{II}}\right)+2\left(\sum_{i=1}^{4}y_{i}{}^{2}{ϒ}_{i}^{\mathrm{III}}+y_{4}{}^{2}{ϒ}_{5}^{\mathrm{III}}\right)$$

For the assumed pdf given by Equation (7), we further derive:

(10) $${ϒ}^{I}=2\sum_{i=1}^{5}\int_{x_{i-1}}^{x_{i}}x^{2}p\left(x\right)dx=\sigma^{2}$$

(11) $${ϒ}_{i}^{\mathrm{II}}=\int_{x_{i-1}}^{x_{i}}xp\left(x\right)dx=\frac{1}{2}\left[\left(x_{i-1}+\frac{\sigma }{\sqrt{2}}\right)\exp\left\{-\frac{\sqrt{2}x_{i-1}}{\sigma }\right\}-\left(x_{i}+\frac{\sigma }{\sqrt{2}}\right)\exp\left\{-\frac{\sqrt{2}x_{i}}{\sigma }\right\}\right],\;i=1,\ldots ,5$$

(12) $${ϒ}_{i}^{\mathrm{III}}=\int_{x_{i-1}}^{x_{i}}p\left(x\right)dx=\frac{1}{2}\left[\exp\left\{-\frac{\sqrt{2}x_{i-1}}{\sigma }\right\}-\exp\left\{-\frac{\sqrt{2}x_{i}}{\sigma }\right\}\right]\;,\;i=1,\ldots ,5$$

Eventually, by substituting Equations (10)–(12) into Equation (9), we derive:

(13) $$D^{\mathrm{UQ}}=\sigma^{2}+{\left(\frac{x_{\max}}{8}\right)}^{2}-\sqrt{2}\sigma \frac{x_{\max}}{8}\left[1+2\sum_{i=1}^{3}\exp\left\{-i\frac{\sqrt{2}x_{\max}}{4\sigma }\right\}\right]$$

Similarly, as in numerous papers about quantization (for instance, in [6,9,22,23,24,25,26,27,28,29,30,32,34,35,36,38,40]), we are interested in conducting an analysis for the variance-matched case, where the designed for and applied to variance of data being quantized match and commonly amount to 1. Therefore, we further assume *σ*^2^ = 1 so that we end up with the following novel closed-form formula for the distortion of the symmetrical three-bit UQ when the input has the Laplacian pdf of zero mean and unit variance:

(14) $$D^{\mathrm{UQ}}|{}_{\sigma^{2}=1}=1+{\left(\frac{x_{\max}}{8}\right)}^{2}-\sqrt{2}\frac{x_{\max}}{8}\left(1+2\sum_{i=1}^{3}\exp\left\{-i\frac{\sqrt{2}x_{\max}}{4}\right\}\right)$$

Let us finally define the theoretical SQNR as:

(15) $${\mathrm{SQNR}}_{\mathrm{th}}^{\mathrm{UQ}}=10\log_{10}\left(\frac{\sigma^{2}}{D^{\mathrm{UQ}}}\right)$$

which will also be calculated for *σ*^2^ = 1 and compared with the experimentally determined SQNR_ex_^UQ^.

## 4. Specification of Two NN Models and Post-Training Prerequisites

**Figure 3:**
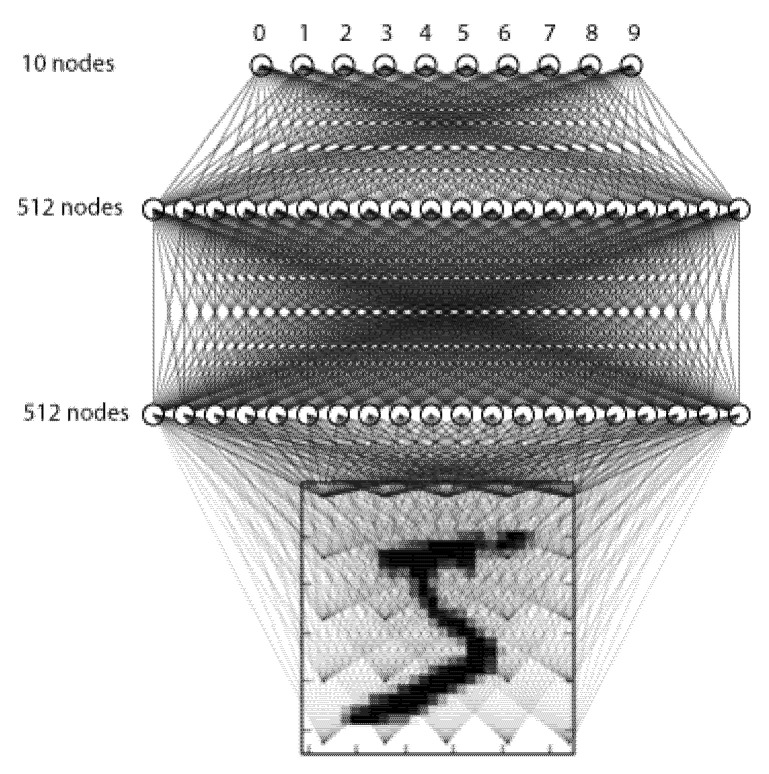
MLP model for handwritten digit recognition.

After we have defined the quantizer, we can proceed with the design and implementation of the two NN model architectures for the practical application of our three-bit UQ in post-training NN quantization. The first chosen NN model architecture is MLP and is fairly simple, consisting of three fully connected layers (see Figure 3), as our goal is to analyze the impact of the quantizer design on the MLP model’s accuracy but not to achieve the highest possible accuracy on the dataset itself. Moreover, our goal is to provide a fair comparison with the results from [34], where the same architecture was assumed. The second chosen NN model architecture is CNN, which contains one additional convolutional layer.

Both NN models have been trained on the MNIST dataset for handwritten digits recognition, consisting of 60,000 black and white images of handwritten digits from 0 to 9 [41]. In particular, the MNIST dataset consists of 70,000 black and white images of handwritten digits, with pixel values in the range [0–255]. The dataset is split into 60,000 training and 10,000 testing sets, while all images have equal dimensions of 28 × 28 pixels [41]. The images for MLP are being flattened into one-dimensional vectors of 784 (28 × 28) elements to match the shape accepted by our first NN, while for a proper CNN input, one additional dimension is being added to represent the channel. The last pre-processing step is normalizing the input into the range [0–1] by dividing every image sample with the highest possible value for black and white image amplitude of 255.

The MLP model consists of two hidden and one output layer, with three fully connected (dense) layers in total (see Figure 3). Hidden layers consist of 512 nodes, while the output layer consists of 10 nodes, where every node estimates the probability that the MLP output is any digit from 0 to 9. Both hidden layers utilize ReLU activation and dropout regularization, which sets 20% of the outputs of hidden layers to 0 to prevent overfitting [42]. In the output layer, the SoftMax activation is utilized, which outputs probabilities that the input belongs to any of 10 possible classes. Training and accuracy evaluation of our MLP model has been implemented in TensorFlow framework with Keras API, version 2.5.0 [43].

The CNN model consists of one convolutional layer, followed by ReLU activation, max pooling and flatten layer, whose output is fed to the previously described MLP with two hidden FC layers and the output layer. The convolutional layer contains 16 filters with kernel size set to 3 × 3, while the max pooling layer utilizes a pooling window of size 2 × 2. The output of the pooling layer is further flattened into a one-dimensional vector for feeding it forward to the FC dense layer. The only difference between the previously described MLP and the dense layers utilized in CNN is in the dropout percentage, which is in the case of CNN set to 50%, to further prevent overfitting of the FC layers. Therefore, the CNN model consists of three hidden layers and the output layer with a total of 1,652,906 trainable parameters. The training is performed on the MNIST, in the same manner as for the MLP, with a total of 10 epochs, while the batch size is equal to 128 training samples.

Additional results are also provided in the paper for both specified models, MLP and CNN, trained on the Fashion-MNIST dataset [44]. Fashion-MNIST is a dataset comprising of 28 × 28 grayscale images of 70,000 fashion products from 10 categories, with 7000 images per category [44]. The training set has 60,000 images and the test set has 10,000 images. Fashion-MNIST shares the same image size, data format and the structure of training and testing splits with the MNIST. It has been highlighted in [45] that although Fashion-MNIST dataset poses a more challenging classification task compared to MNIST dataset, the usage of MNIST dataset does not seem to be decreasing. Additionally, it has been pointed out at the fact that the reason MNIST dataset is still widely utilized comes from its size, allowing deep learning researchers to quickly check and prototype their algorithms.

For both NNs, training, accuracy analysis, and quantization have been implemented in the Python programming language [46]. After training NN models, the corresponding (different) weights are stored as 32-bit floating points, which represents full precision in the TensorFlow framework. The MLP model achieves the accuracy of 98.1% on the MNIST validation set and 88.96% on the Fashion-MNIST validation set, obtained after 10 epochs of training. The CNN model achieves a higher accuracy of 98.89% on the MNIST validation set and 91.53%, obtained on the Fashion-MNIST validation set, also after 10 epochs of training.

The first NN model (MLP) consists of 669,706 parameters, which are all fed to our three-bit UQ after training. As mentioned, the second NN model (CNN) consists of 1,652,906 parameters, which are also all fed to our three-bit UQ after training in the CNN case. While compressing the model weights, we reduce the bit rate for every parameter from 32 to 3 bits per sample. Once we have obtained the quantized representations of the NN model weights, we can feed them back into the model and assess the QNN model performance when the post-training quantization is applied, and each of the NN weights is represented with only 3 bits. This holds for both NNs addressed in this paper.

In brief, to evaluate the performance of our three-bit UQ in practice, we have conducted an experimental training of two NN models, and we have stored the corresponding weights represented in FP32 format. As most machine learning frameworks store trained weights as 32-bit floating-point values, this bit rate is treated as baseline (full) precision. After the two training processes were complete, we accessed the stored weights, applied the quantization, and loaded the quantized weights into the corresponding models. As previously stated, by doing so, we can significantly reduce the amount of storage required for saving the QNN models. As in any lossy compression task, where the original input values cannot be restored after the compression/quantization is applied, an irreversible quantization error is introduced, which can have a negative impact on the accuracy of our models. However, by representing the weights of MLP and CNN by 3 bits per sample, we perform compression with a factor larger than 10 times. When performing compression of that amount, it is crucial to make the best use out of available resources, which in this case is our available bit rate.

Typically, when performing uniform quantization of nonuniform unrestricted sources, e.g., the Laplacian source, the choice of the support region width (ℜ*_g_* width) is the most sensitive quantizer parameter, which can significantly impact the performance [22,23,24,25,30,31,35]. We have already confirmed this premise for the bit rate of 2 bits per sample in [34], where the support region choice is crucial not only for the performance of the quantizer alone but also for the QNN model’s accuracy due to only four representation levels being available. This imposed a question of whether the choice of ℜ*_g_* will have an equally strong impact on SQNR and accuracy for the case of 3 bits per sample available, which will be discussed in the later sections. Let us highlight that to provide a fair comparison with the results from [34], in this paper, the identical MLP architecture is assumed, and the identical weights (stored in FP32 format) are uniformly quantized by using one additional bit. Essentially, we evaluate and compare the performance of multiple three-bit UQ designs, where we also highlight a few significant cases of its design to provide a fair comparison with the results from [34]. By doing so, we aim to determine the influence of the choice of the key parameter of the three-bit UQ on the performance of both quantizer and QNN models, whereas, compared to the analysis of the numerical results from [34], the analysis presented in this paper is much wider. Moreover, unlike [34], where the analysis has been only performed for MLP and MNIST datasets, in this paper, the analysis is additionally performed for a CNN model and the MNIST dataset. Eventually, in this paper, results are also provided for both specified models, MLP and CNN, trained on the Fashion-MNIST dataset [44].

## 5. Post-Training Three-Bit Uniform Quantization

As previously mentioned, compression of NNs in various QNN scenarios implies the reduction in the number of weights representations while targeting the smallest possible accuracy loss. Some QNNs apply weights quantization in the training phase and provide the recovery of QNN accuracy to some extent through the retraining phase. In contrast, in this paper, due to simplicity reasons as well as applicability reasons, especially for resource-constrained devices, we want to benefit from weights quantization in the post-training phase and to avoid a retraining phase. With given pre-trained NN models, MLP and CNN, the main goal that we set to the UQ in question is to compress the original FP32 weights to three-bit representations and to provide accuracy preservation. Moreover, for both NNs, we examine whether the key parameter of the observed UQ has a strong impact on accuracy preservation. In the following, we describe the post-training quantization procedure (see our Algorithm 1), incorporating three sequential operations applied to NN weights: normalization, quantization, and denormalization.
**Algorithm 1** Weights compression by means of post-training quantization using the symmetrical three-bit UQ**Notation:***w_j_*—pretrained weight, *w_j_*^N^—normalized weight, *w_j_*^UQN^—uniformly quantized normalized weight, *w_j_*^UQ^—uniformly quantized weight**Input: *Ŵ*** = {*w_j_*}*_j_*_=1, 2, …, *W*_, weights represented in FP32 format**Output: *Ŵ***^UQ^ = {*w_j_*^UQ^}*_j_*_ = 1, 2, …, *W*_, uniformly quantized weights, ${\mathrm{SQNR}}_{\mathrm{th}}^{\mathrm{UQ}}$, ${\mathrm{SQNR}}_{\mathrm{ex}}^{\mathrm{UQ}}$, Accuracy
 1: loading initial pretrained weights ***Ŵ*** = {*w_j_*}*_j_*_= 1, 2, …, *W*_ 2: calculating mean(***Ŵ***) and std(***Ŵ***) 3: normalization of weights by using (16) 4:  forming ***Ŵ*^N^** = {*w_j_*^N^}*_j_*_= 1, 2, …, *W*_ 5: *w*_min_← minimal value of the normalized weights from ***Ŵ*^N^** 6: *w*_max_ ← maximal value of the normalized weights from ***Ŵ*^N^**
 7: Choosing *w*_supp_//*w*_supp_ ←|*w*_min_| or *w*_max_ or *x*_max_[J] or *x*_max_[H] or some other given value 8: *j* ← 1 9:  **while**
*j* ≤ *W*
**do** 10:   quantization of *w_j_*^N^ by using (17) 11:   **end while** 12:   denormalization of quantized weights  13:   forming ***Ŵ*^UQ^** 14:   invoking (18) to calculate $D_{\mathrm{ex}}^{\mathrm{UQ}}$ 15:   calculating ${\mathrm{SQNR}}_{\mathrm{ex}}^{\mathrm{UQ}}$ by using (19) 16:   calculating ${\mathrm{SQNR}}_{\mathrm{th}}^{\mathrm{UQ}}$ by using (15) 17:   estimating Accuracy 18:   **return *Ŵ***^UQ^, ${\mathrm{SQNR}}_{\mathrm{th}}^{\mathrm{UQ}}$, ${\mathrm{SQNR}}_{\mathrm{ex}}^{\mathrm{UQ}}$, Accuracy

The normalization of weights is the first operation, applied to all weights represented by the one-dimensional vector ***Ŵ*** = {*w_j_*}*_j_*_= 1, 2, …, *W*_ of *W* elements, where *W* = 669,706 is the total number of weights we take into the account in the MLP case. Each weight is normalized, thereby forming the vector ***Ŵ*^N^** = {*w_j_*^N^}*_j_*_=1, 2, …, *W*_

(16) $$w_{j}^{N}=\frac{w_{j}-\mathrm{mean}(\hat{W})}{\mathrm{std}(\hat{W})}$$

where mean(***Ŵ***) and std(***Ŵ***) are the mean and the standard deviation of values from the vector ***Ŵ***, respectively. In brief, weights represented in the FP32 format are normalized to have zero mean and unit variance before feeding them in the quantization process. Once the weights are normalized, a discrete distribution of normalized weights is formed with *w*_min_ and *w*_max_ denoting the minimal and the maximal value of the normalized weights, where the notation N indicating the normalization procedure is here omitted due to simplicity reasons.

The second step in our post-training quantization of NN weights is applying three-bit uniform quantization to the normalized weights. By the quantization procedure, all normalized weights are divided into two complementary sets: the weights falling in and the weights falling out of the support region [−*w*_supp_, *w*_supp_]. In quantization, the support region is typically specified so as to cover a very large number of weights. For a given *w*_supp_, we perform uniform quantization of the normalized weights by using

(17) $$w_{j}^{\mathrm{UQN}}=Q_{w_{j}^{N}}^{\mathrm{UQ}}\left(w_{j}^{N};w_{\mathrm{supp}}\right)=\left\{ \begin{array}{l} \mathrm{sgn}(w_{j}^{N})\left(\left\lfloor \frac{4\left|w_{j}^{N}\right|}{w_{\mathrm{supp}}}\right\rfloor +\frac{1}{2}\right)\frac{w_{\mathrm{supp}}}{4},\;\left|w_{j}^{N}\right|\leq w_{\mathrm{supp}}\\ \mathrm{sgn}(w_{j}^{N})\cdot \frac{7w_{\mathrm{supp}}}{8},\;\left|w_{j}^{N}\right|>w_{\mathrm{supp}} \end{array}\right.$$

thereby forming ***Ŵ***^UQN^ = {*w_j_*^UQN^}*_j_*_= 1, 2, …, *W*_, whose values can take up to 8 different values.

The last step in the post-training procedure is the denormalization of the quantized weights. Denormalization is performed after quantization so that quantized weights being denormalized, ***Ŵ***^UQ^ = {*w_j_*^UQ^}*_j_*_=1,2,…,*W*_, could go back to the original range of values and could be loaded into the QNN model.

Eventually, we can evaluate the performance of the three-bit UQ measured with distortion or SQNR

(18) $$D_{\mathrm{ex}}^{\mathrm{UQ}}=\frac{1}{W}{\left\|\hat{W}-{\hat{W}}^{\mathrm{UQ}}\right\|}_{2}^{2}=\frac{1}{W}{\left(\hat{W}-{\hat{W}}^{\mathrm{UQ}}\right)}^{T}\left(\hat{W}-{\hat{W}}^{\mathrm{UQ}}\right)=\frac{1}{W}\sum_{j=1}^{W}{\left(w_{j}-w_{j}^{\mathrm{UQ}}\right)}^{2}$$

(19) $${\mathrm{SQNR}}_{\mathrm{ex}}^{\mathrm{UQ}}=10\log_{10}\left(\frac{\frac{1}{W}\sum_{j=1}^{W}w_{j}^{2}}{D_{\mathrm{ex}}^{\mathrm{UQ}}}\right)$$

and we can estimate the accuracies of both QNNs (for MLP and CNN), which also reflect the performance of the post-training three-bit UQ.

## 6. Numerical Results and Analysis

The performance analysis of our post-training quantization can be broadly observed in two aspects: the accuracy of the QNN models and the SQNR of the uniform quantizers implemented to perform the weight compression. Our main goal is to determine and isolate the impact of the choice of ℜ*_g_* on the accuracy of the QNNs. Therefore, for the first NN model (MLP), we have conducted a detailed analysis of the statics of both the original and the uniformly quantized weights. For both NN architectures, MLP and CNN, which are trained on the MNIST dataset, we have determined the accuracies of the QNNs for various values of the key quantizer parameter for the bit rate of *R* = 3 bit/sample. As previously mentioned, the full precision accuracy of our MLP on the MNIST validation dataset amounts to 98.1%, which is the benchmark accuracy used in the comparisons. The second point of reference is the accuracy of the QNN presented in [34], where we have obtained accuracy up to 96.97% with the use of two-bit UQ for the quantization of identical weights with the identical MLP architecture. Let us highlight that we have shown in [34] that an unsuitable choice of the support region can significantly degrade the accuracy (more than 3.5%), that is, the performance of the post-training two-bit uniform quantization for the assumed MLP model trained on MNIST. The second point of reference will give us insight into the differences in impact of ℜ*_g_* on the QNN accuracy when using 1 bit per sample more for compression. The intuition is that for a larger bit rate of *R* = 3 bit/sample, the influence of the support region of the quantizer will be smaller compared to the case presented in [34], where only 2 bits have been used to represent the QNN weights. To confirm this premise, we have designed multiple three-bit UQs with different ℜ*_g_* values and analyzed the accuracy accordingly, which will be presented later in this section.

Firstly, we have conducted multiple training procedures of the specified MLP on the MNIST dataset to confirm that in various training sequences, the weights converge to the values that belong to the same range while obtaining similar model accuracy for both MLP and quantized MLP models. Table 1 shows the statistics of the trained weights and the performance of our MLP model without and with the application of quantization for four independent training procedures of the same MLP model on the MNIST dataset. One can observe that the weights fall into almost the same range of values for each training, with and without normalization. Additionally, the model achieves similar accuracy throughout various training procedures with small variations. Finally, the obtained SQNR values (for the case where the support region threshold is specified by (6)) are very close for different weight vectors, with dynamics of approximately 0.04 dB. We can conclude that the weight vector chosen in our analysis (the same weight vector as utilized in [34]) can be considered a proper representation of any training sequence obtained with the same MLP architecture and that the results of post-training quantization are very close to each other for all of the observed cases.

Once we have established that the chosen weights represent a good specimen for any training sequence of a given MLP architecture, we can proceed with the QNN accuracy evaluation. Table 2 presents SQNR of the three-bit UQ and QNN model accuracy, obtained for multiple choices of ℜ*_g_* for the bit rate of *R* = 3 bit/sample and the specified MLP trained on MNIST dataset. Along with the experimentally obtained SQNR values (calculated by using Equations (18) and (19)), we have determined theoretical SQNR by using Equations (14) and (15), for each ℜ*_g_* choice to evaluate differences between the theoretical and experimental SQNR values. Choices 1 and 2 depend on the statistics of the normalized NN model weights, specifically on the minimum and maximum weight value (*w*_min_ and *w*_max_), while in Choice 3 and 4, ℜ*_g_* is specified with the well-known optimal and asymptotically optimal values for the assumed bit rates, as given in [22,31]. In addition, Table 3 presents SQNR and the QNN model’s accuracy obtained with the application of two-bit UQ, where ℜ*_g_* is defined following the same principles as in Table 2 and the identical weights of the MLP model (trained on MNIST dataset) are quantized [34]. Specifically, Choice 1 of ℜ*_g_* in Table 2 matches Case 1 in Table 3, etc. Table 3 is provided for comparison purposes to help us make conclusions about the different impacts that the choice of ℜ*_g_* has on the SQNR and QNN model’s accuracy for a specified MLP and different bit rates. It has been highlighted in [47] that Choice 2, that is, Case 2, is a popular choice for the quantizer design, which, as one can notice from Table 3, is not the most suitable choice in terms of accuracy and SQNR. In the following, we first analyze the results obtained for the MLP and the three-bit UQ presented in Table 2, with a reference to the two-bit uniform quantization model presented in [34].

Choice 1 defines ℜ*_g_* of the UQ in the range [−*w*_max_, *w*_max_], meaning that ℜ*_g_* is symmetrically designed according to the absolute value of the maximum weight sample. In our case with the MLP, it holds |*w*_max_| = *w*_max_ (*w*_max_ ≥ 0), so we have simplified our notation. From Table 2 and Table 3, one can notice that with the so-defined ℜ*_g_*, 99.988% of the normalized weight samples are within ℜ*_g_*. Choice 2 defines ℜ*_g_* in the range [*w*_min_, −*w*_min_], meaning that our quantizer is symmetrically designed according to the absolute value of the minimum normalized weight sample, |*w*_min_|. In our case, it holds |*w*_min_| = −*w*_min_ (*w*_min_ ≤ 0). ℜ*_g_* defined in Choice 2 includes all of the normalized weight samples, meaning that none of the samples fall into the overload region of the quantizer. From Table 2, one can notice that although SQNR for Choices 1 and 2 differ to a great extent, the QNN model’s accuracy is identical. In contrast, one can notice from Table 3 that with two-bit UQ, the QNN model exhibits significant performance variations in both SQNR and model accuracy, where both are dominantly determined by the choice of ℜ*_g_*. Similar is the case with Choices 3 and 4, where *x*_max_[H] and *x*_max_[J] take relatively close values. Although SQNR obtained for Choices 3 and 4, presented in Table 2, and the matching Cases 3 and 4 from Table 3 has stable, close values, this is not the case for the QNN model’s accuracy (see Table 3).

By analyzing the QNN model accuracies presented in Table 2, we can observe that the accuracy dynamics, defined as the difference between the highest and lowest obtained accuracy, amounts to 0.2%. On the other hand, by performing the same analysis for the case of implementing two-bit UQ, we can calculate that QNN accuracy dynamics amounts to 2.39%. Moreover, by comparing the SQNR values obtained for the three-bit UQ presented in Table 2 for Choices 2 and 4, we can observe a high dynamic range of SQNR values with the difference between Choices 2 and 4 amounting to 6.3146 dB, while the difference in the QNN model’s accuracy for these choices of ℜ*_g_* amounts to just 0.2%. This further indicates that a large difference in SQNR of the three-bit UQ does not necessarily result in a large difference in the accuracy of the QNN, as had been found for the two-bit UQ in [34]. The maximum theoretical and experimental SQNR is obtained for Choice 4, where ℜ*_g_* is defined optimally by *x*_max_[J], while the maximum QNN model accuracy is obtained in Choices 1 and 2 (see bolded values). We can conclude that the choice of ℜ*_g_* dominantly determines the QNN model’s accuracy in the case of a very low bit rate of 2 bits per sample, where we have only four available representation levels. By doubling the number of representation levels to eight for a bit rate of *R* = 3 bit/sample, it turns out that the impact of the ℜ*_g_* on QNN model’s accuracy significantly reduces. This statement will be inspected later in this section by observing the QNN model’s accuracy in a wider range of ℜ*_g_* choices for both MLP and CNN models.

**Figure 4:**
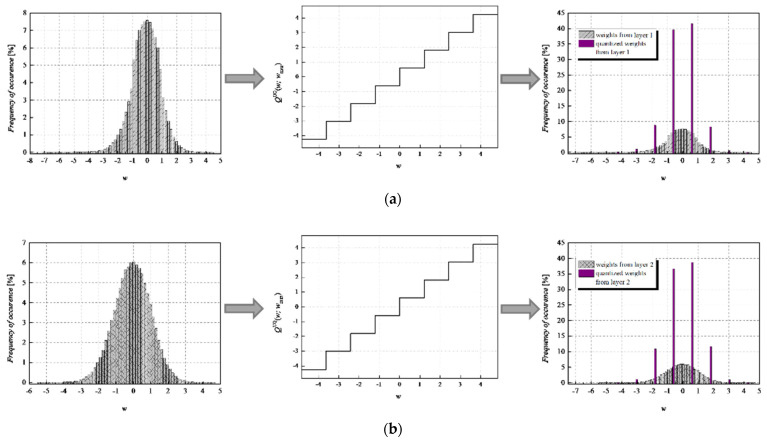

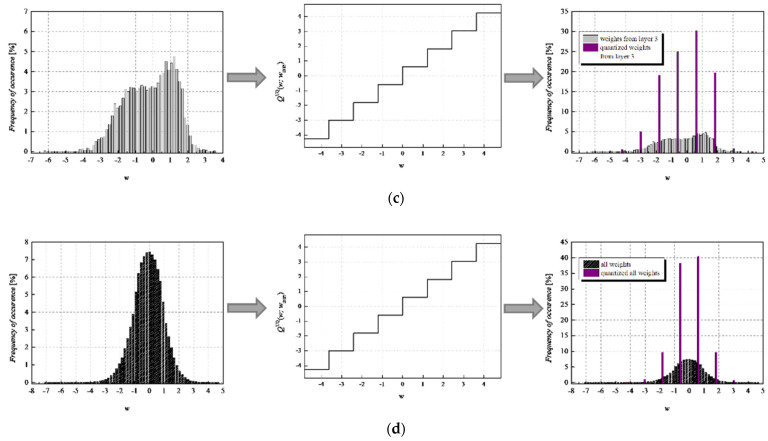
Normalized histogram of weights (FP32) for MLP model trained on MNIST dataset from (**a**) layer 1, (**b**) layer 2, (**c**) layer 3, and (**d**) all layers; Transfer characteristic of the symmetric three-bit UQ for the ℜ*_g_* Choice 1; Normalized histogram of FP32 and uniformly quantized weights from (**a**) layer 1, (**b**) layer 2, (**c**) layer 3, and (**d**) all layers of MLP.

**Figure 5:**
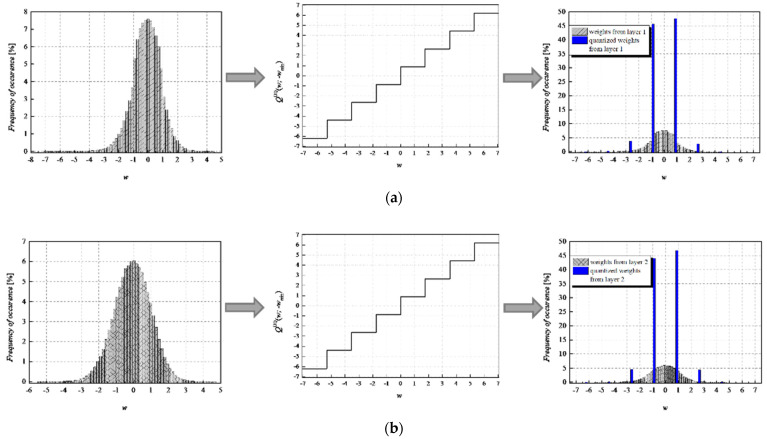

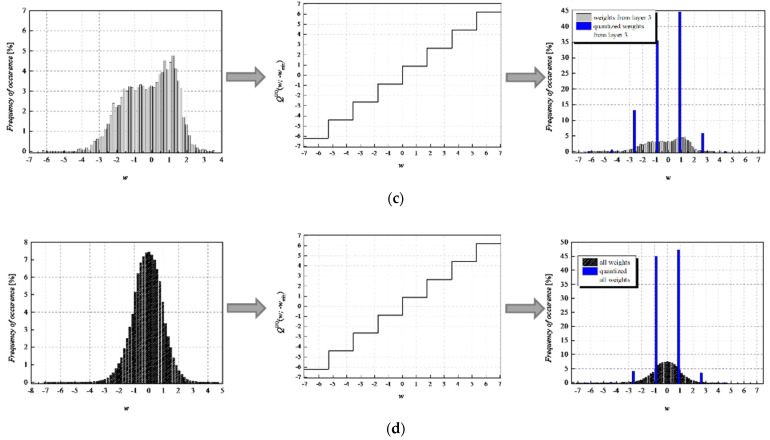
Normalized histogram of weights (FP32) for MLP model trained on MNIST dataset from (**a**) layer 1, (**b**) layer 2, (**c**) layer 3, and (**d**) all layers; Transfer characteristic of the symmetric three-bit UQ for the ℜ*_g_* Choice 2; Normalized histogram of FP32 and uniformly quantized weights from (**a**) layer 1, (**b**) layer 2, (**c**) layer 3, and (**d**) all layers of MLP.

**Figure 6:**
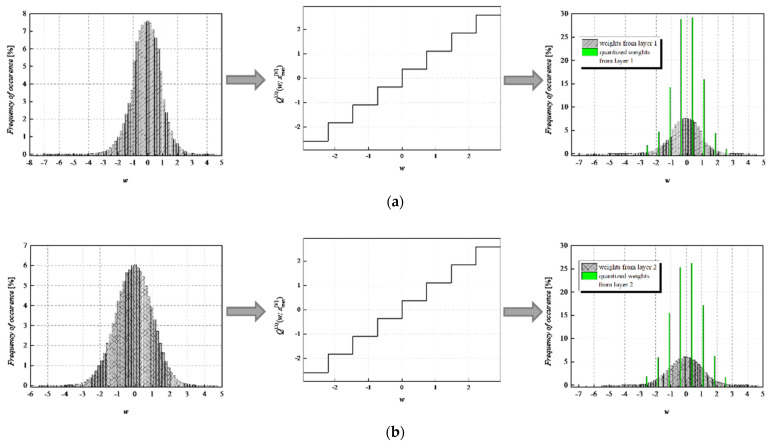

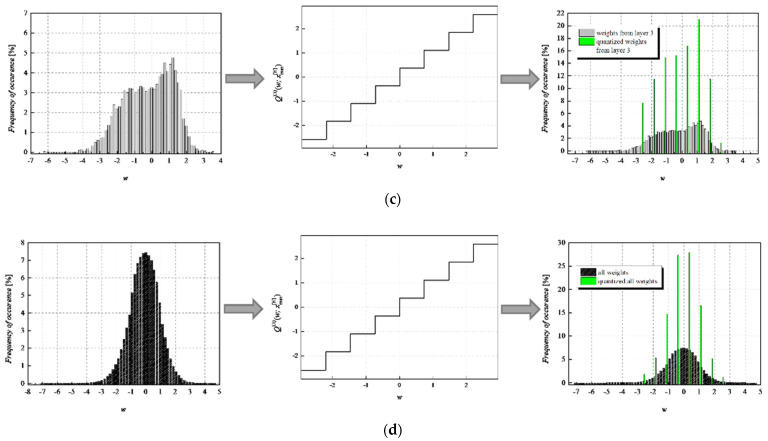
Normalized histogram of weights (FP32) for MLP model trained on MNIST dataset from (**a**) layer 1, (**b**) layer 2, (**c**) layer 3, and (**d**) all layers; Transfer characteristic of the symmetric three-bit UQ for the ℜ*_g_* Choice 3; Normalized histogram of FP32 and uniformly quantized weights from (**a**) layer 1, (**b**) layer 2, (**c**) layer 3, and (**d**) all layers of MLP.

**Figure 7:**
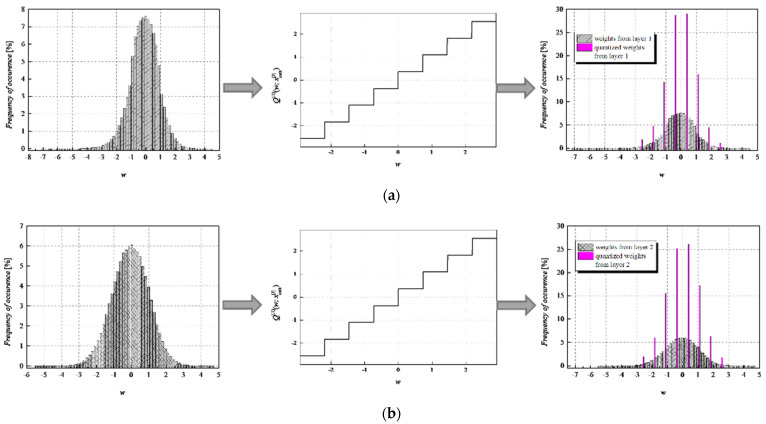

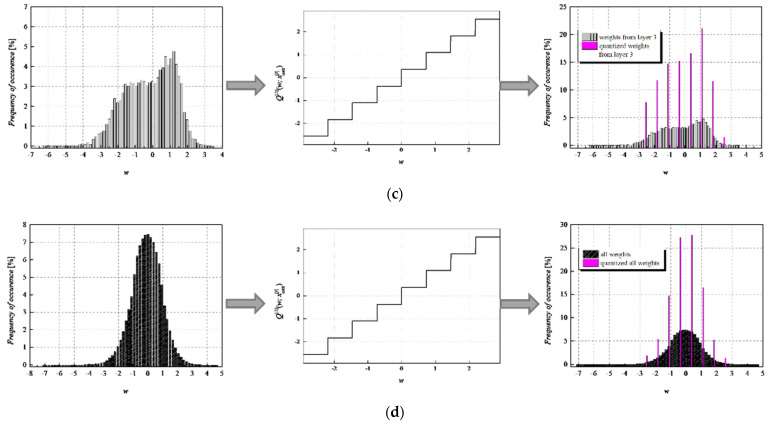
Normalized histogram of weights (FP32) for MLP model trained on MNIST dataset from (**a**) layer 1, (**b**) layer 2, (**c**) layer 3, and (**d**) all layers; Transfer characteristic of the symmetric three-bit UQ for the ℜ*_g_* Choice 4; Normalized histogram of FP32 and uniformly quantized weights from (**a**) layer 1, (**b**) layer 2, (**c**) layer 3, and (**d**) all layers of MLP.

To achieve a high-quality compressed signal, it is of great significance to exploit the full potential of the available bit rate. To inspect the distribution of the normalized weights among different representation levels of the three-bit UQ, we have extracted histograms of normalized weights of individual layers, as well as the joint histogram of all normalized weights of the MLP (trained on MNIST dataset) before and after compression/quantization (see Figure 4, Figure 5, Figure 6 and Figure 7). At first glance, by comparing Choices 1 and 2 with Choices 3 and 4, one can notice that in the first two choices, we dominantly utilize only four of the eight available representation levels of UQ, which is especially noticeable on the histogram presenting all normalized weights of QNN model for Choice 2. This leads us to the conclusion that in Choices 1 and 2, we utilize unnecessary wide ℜ*_g_*, which explains the lower SQNR values obtained for the first two choices, especially in Choice 2, where ℜ*_g_* ranges [−7.063787, 7.063787]. At the same time, a wider ℜ*_g_* positively contributes to the QNN model’s accuracy, which is higher for the first two observed choices compared to Choices 3 and 4.

By observing the histograms of Choices 3 and 4, one can notice that all representation levels of the three-bit UQ take enough values to be clearly noticeably on the QNN histogram, implying that during the quantization process, we exploit the full potential of the three-bit UQ. Moreover, this can be confirmed by comparing the histogram envelope of all normalized weights before and after quantization. One can notice that in Choices 3 and 4, the envelope of the histogram of the quantized normalized weights follows the envelope of the histogram of all normalized weights before quantization to a great extent. This shows us that the distribution of the quantized normalized weights follows the distribution of the normalized weights. Additionally, by analyzing the normalized weights in different NN model layers, we can see that level 3 has the highest deviation from the Laplacian distribution, having a distribution that is closer to the uniform one. As the uniform quantizer is optimal for the uniform distribution, by making a suitable choice of ℜ*_g_*, the benefits of the UQ model can be significantly exploited for this layer. Histograms offer us another interesting insight that lies in a small asymmetry of the QNN model normalized weights. This phenomenon is a consequence of the fact that the percentage of non-negative weights after normalization is 50.79%, meaning that it is higher by 1.58% than the percentage of negative weight values after normalization. This leads to a righthand side shift of the samples during quantization, which can be observed in individual and joint histograms of normalized weights before and after quantization. The asymmetry is largest at layer 2, where the percentage of positive weight samples after normalization amounts to 51.3649%, meaning that it is higher by 2.7298% than the percentage of negative weight values after normalization. As a result, we have the highest level of asymmetry after quantization at layer 2.

**Figure 8:**
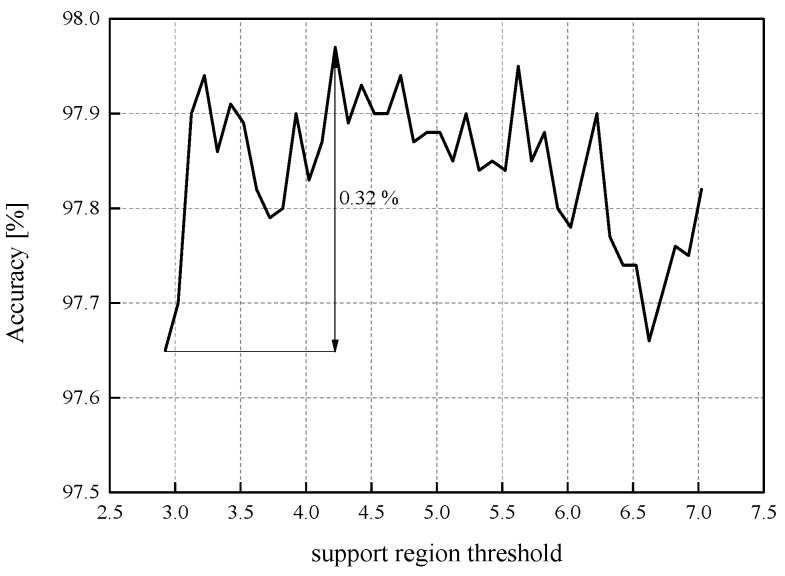
The accuracy of the first QNN model (MLP trained on MNIST dataset) for *w*_supp_ ranging from *x*_max_[J] = 2.9236 to |*x*_min_| = 7.063787.

So far, we have confirmed that in all four observed choices of ℜ*_g,_* our first QNN model achieves a stable performance in terms of accuracy, with a deviation of approximately 0.2%. To further inspect the influence that *w*_supp_ has on the QNN model’s accuracy, we have determined the QNN model’s accuracy for *w*_supp_ values in the range from *x*_max_[J] = 2.9236 to |*x*_min_| = 7.063787, with a step size of 0.1 (see Figure 8). The highest obtained accuracy in the observed range amounts to 97.97%, while the lowest value is 97.65%, that is, the accuracy dynamics amounts to 0.32%. Therefore, we have once more confirmed that for a bit rate of *R* = 3 bit/sample, the impact of the ℜ*_g_* choice on the QNN model’s accuracy significantly reduces compared to the case where the identical weights of MLP, stored in FP32 format, are uniformly quantized with the bit rate of 2 bit/sample. It should be mentioned out that accuracy dynamics presented in Figure 8 has been determined in a wide but carefully chosen range of the observed values of *w*_supp_, which we heuristically specified.

**Figure 9:**
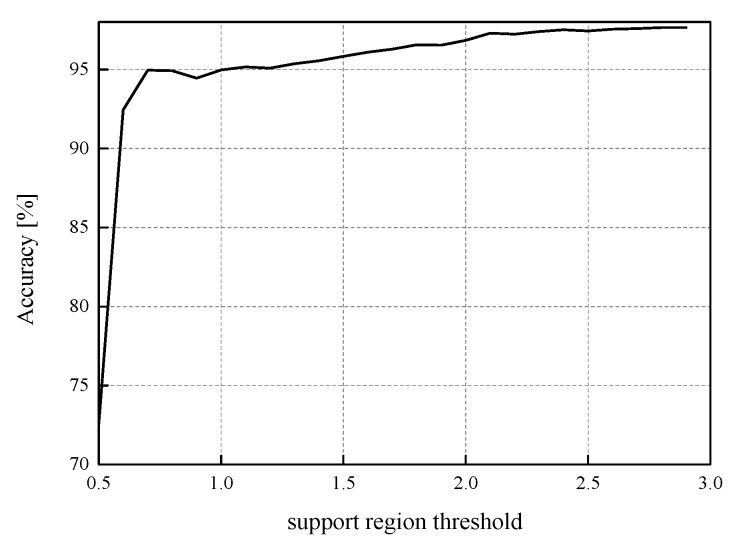
The accuracy of the first QNN model (MLP trained on MNIST dataset) for partially unfavorable choice of the support region threshold.

In this paper, we also present a range of unfavorable ℜ*_g_* choices for MLP, i.e., the first NN model observed (see Figure 9). If the support region threshold values range from 0.5 to *x*_max_[J] = 2.9236 and change with a step size of 0.1, the QNN’s accuracy varies from 72.51% to 97.65%. As this presents very high dynamic values, we can conclude that all four choices presented in Table 2 have been carefully made according to the nature of our input data. In addition, it is noticeable that after passing the value of 2.5, the accuracy is persistently above 97% due to a small impact of different ℜ*_g_* choices on the QNN model’s accuracy if *w*_supp_ is chosen from a proper intuitively expected range, that is when it holds *w*_supp_ ≥ 2.5.

**Figure 10:**
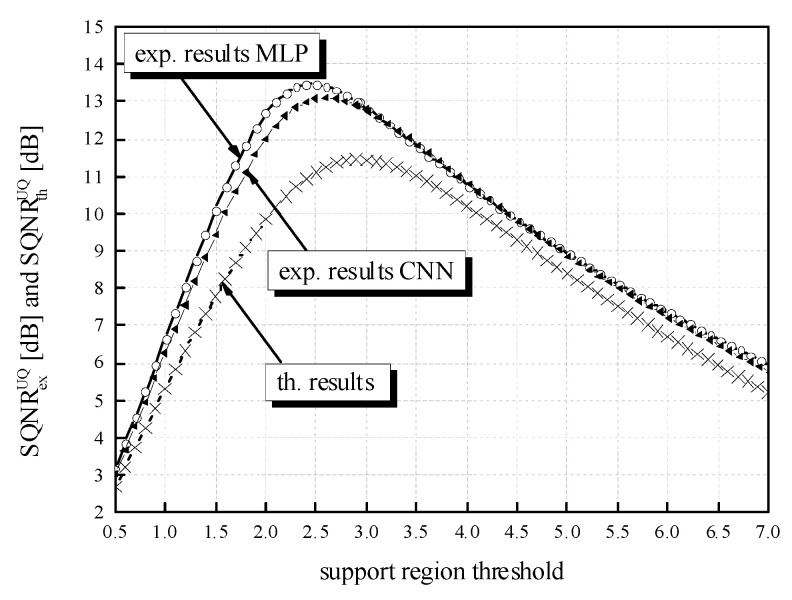
Theoretical and experimental SQNR values for a wide range of *w*_supp_ values assumed in post-training three-bit UQ for MLP and CNN trained on MNIST dataset.

**Figure 11:**
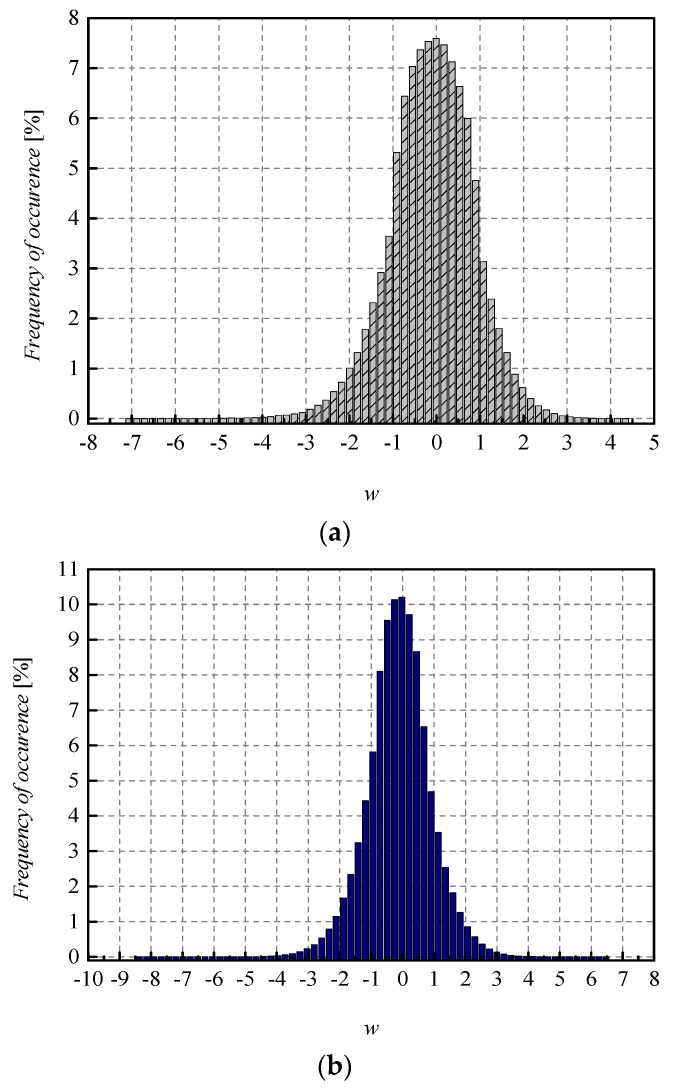
Normalized histogram of weights (FP32) for (**a**) MLP (**b**) CNN, both trained on MNIST dataset.

While the QNN model’s accuracy is the main point of reference observed in the experiments, the SQNR of the three-bit UQ takes a significant place in our analysis. Similar to the evaluation of the QNN model’s accuracy dynamics, we can observe the impact that the choice of ℜ*_g_* has on the performance of the three-bit UQ. Table 2 already showed that for a bit rate of *R* = 3 bit/sample, SQNR values have much higher dynamics compared to the QNN model’s accuracy. To inspect the impact of *w*_supp_ choice on the SQNR in a wider range of values, we have calculated the theoretical and experimental SQNR of the three-bit UQ in the *w*_supp_ range of [0.5, 7.063787]. In addition, to compare experimentally determined values for the two different NN models specified in the paper, MLP and CNN, in Figure 10, we provide additional experimental results for the CNN model. If we set *w*_supp_ between *x*_max_[J] = 2.9236 and |*x*_min_| = 7.063787 with the step size of 0.1 and calculate the SQNR in 42 points, the dynamics of the experimentally calculated SQNR for the MLP is 12.9766 dB − 5.9005 dB = 7.0761 dB, which is considered to be a very large value. For the values of *w*_supp_ in the range between 0.5 and *x*_max_[J] = 2.9236 with the step size of 0.1, the dynamics of SQNR for the same MLP case is 13.47669 dB − 3.18492 dB = 10.2918 dB. It is obvious that the choice of *w*_supp_ has a large influence on the obtained SQNR of the three-bit UQ. Slightly smaller but also large dynamics of the experimentally calculated SQNR can be perceived for the CNN case. In addition, Figure 10 depicts the difference in theoretically and experimentally obtained SQNR values. One can notice from Figure 10 that the theoretical SQNR curve decreases after passing the theoretically optimal value of *x*_max_[J] = 2.9236. In contrast, the experimentally optimal value of *w*_supp_ is lower compared to the theoretical one. This is a consequence of lower amplitude dynamics of real normalized weights (see Figure 11) used in the experimental analysis compared to the theoretically assumed ones. The maximum of experimentally obtained SQNR values in the case of MLP amounts to 13.47669 dB for the support region threshold equal to 2.5, while the experimentally obtained QNN model accuracy for this ℜ*_g_* choice is 97.43%, which is not the maximum obtained accuracy in the experiment. The maximum of experimentally obtained SQNR value in the CNN case amounts to 13.10203 dB, for the support region threshold equal to 2.6, while the experimentally obtained QNN model accuracy for this ℜ*_g_* choice amount to 98.27%, which is also not the maximum obtained accuracy in the experiment for the CNN case. One can notice the higher amplitude dynamics of weights for the CNN case compared to the MLP case (see Figure 11). For that reason, the maximum of experimentally obtained SQNR values in the CNN case is shifted right in Figure 10. Moreover, one can notice that the theoretically determined SQNR curve is below both experimentally determined SQNR curves. The reason is that in the experimental analysis, the weights originating from the Laplacian-like distribution being quantized are from the limited set of possible values [−7.063787, 4.8371024] for the MLP model and from [−8.372064, 6.469376] for the CNN model. In the theoretical analysis, the quantization of values from an infinite set of values from the Laplacian source is assumed causing an increase in the amount of distortion, that is, the decrease in the theoretical SQNR value. Due to a similar reason (a wider amplitude dynamics of weights in the CNN case compared to the MLP case), the experimentally obtained SQNR is lower in the CNN case in comparison to the MLP case.

When compared to Case 1 of two-bit UQ presented in [34], our three-bit UQ in the comparable case (Choice 1 for the MLP model) achieves a higher accuracy on the MNIST validation dataset by a significant 0.88%. Let us anticipate here that the QNN analysis with UQ should be limited to the cases of bit rate equal to 2 and 3 bits per sample, as it is expected that further increase in the bit rate will not contribute to the significant increase in the QNN model’s accuracy. In other words, we have shown that with the observed three-bit UQ, the accuracy of QNN amounting up to 97.97% can be achieved, meaning that by applying the described post-training quantization procedure to the MLP model trained on the MNIST dataset, the accuracy degradation of only 98.1% − 97.97% = 0.13% can be achieved compared to baseline MLP model stored in FP32 format.

In the following, we analyze the performance of the three-bit UQ in CNN model compression and the impact of various support region threshold values on both SQNR and post-training quantization accuracy. The observed cases and methodologies are the same as previously applied in MLP analysis. In particular, four specific choices of ℜ*_g_* have been observed, and for each choice, we have calculated SQNR and the accuracy of CNN model when three-bit uniform post-training quantization is applied (see Table 4).

One can notice that the CNN model’s accuracy is relatively stable, especially considering very high *w*_min_ and *w*_max_ values, compared to Choices 3 and 4. Moreover, the full precision of the CNN is expectedly higher when compared to the MLP, achieving accuracy of 98.89% on the MNIST validation set. Unlike the accuracy, the SQNR performance of the three-bit UQ differs a lot, which is a result of high absolute *w*_min_ and *w*_max_ values that are very far from the optimal value. Nevertheless, as the fluctuation of the accuracy is smaller than 1%, (98.56% − 97.64% = 0.92%) for such different Choices of ℜ*_g_*, this confirms that the observations made for MLP regarding the influence of ℜ*_g_* on the QNN performance are similar for the case of CNN.

**Figure 12:**
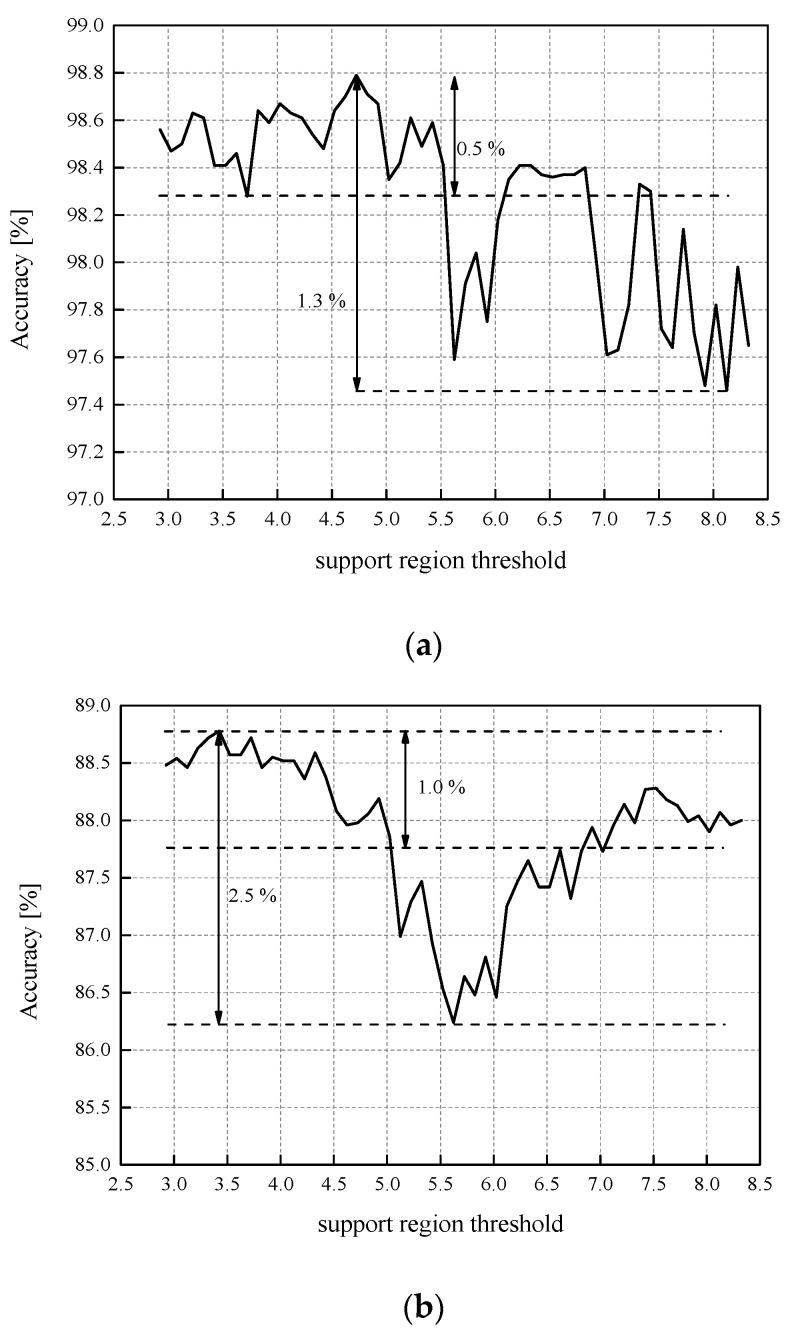

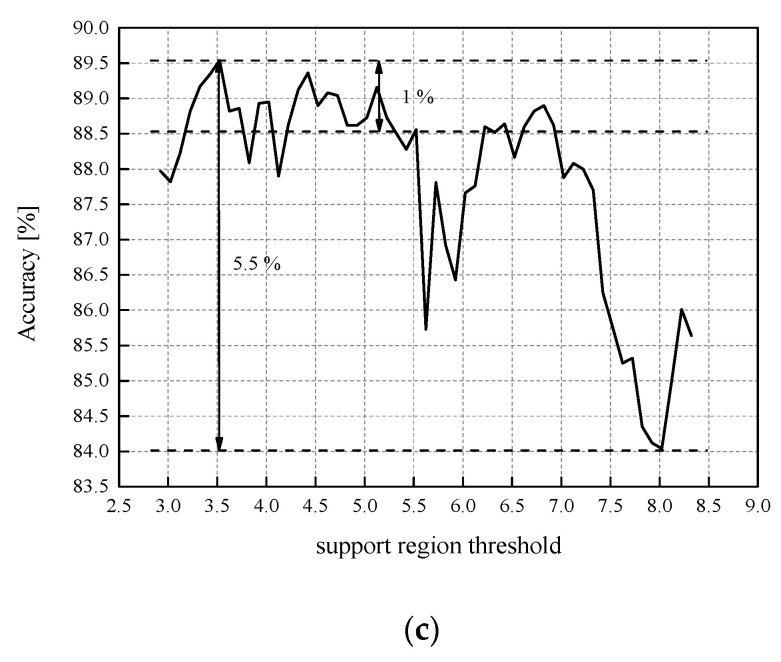
Accuracy after three-bit uniform quantization for (**a**) CNN model trained on MNIST dataset; (**b**) MLP model trained on Fashion-MNIST dataset; (**c**) CNN model trained on Fashion-MNIST dataset—for *w*_supp_ in a wide range from *x*_max_[J] = 2.9236 to |*x*_min_| = 8.372064.

By analyzing the QNN model accuracies presented in Figure 12a for the CNN case, we can observe that the accuracy dynamics, defined as the difference between the highest and lowest obtained accuracy in a very wide range of values selected for the support region threshold (wider than in the MLP case), amounts to 1.3%. On the other hand, by performing the rough analysis based only on the results from Table 3, for the case of implementing two-bit UQ, we can calculate that QNN accuracy dynamics is higher and amounts to 2.39%. At first glance, by comparing the values of SQNR_ex_^UQ^ for MLP and of SQNR_ex_^UQ^ for CNN, for all four choices (see Table 2 and Table 4), one can notice that they have quite similar values for Choices 3 and 4. That is, one can notice that it holds SQNR_ex_^UQ^(MLP- Choices 3 and 4) ≈ SQNR_ex_^UQ^(CNN—Choices 3 and 4). However, for Choices 1 and 2, it is expected that SQNR_ex_^UQ^ (MLP—Choices 1 and 2) > SQNR_ex_^UQ^ (CNN- Choices 1 and 2) due to the higher amplitude dynamics of weights of the CNN model trained on the MNIST dataset compared to the one for the MLP model. Further, by analyzing the percentage of samples belonging to the ℜ*_g_* for the CNN and MLP cases, one can notice that these percentages are quite similar for Choices 1 and 2 (see Table 2 and Table 4—within ℜ*_g_* (%) (MLP—Choices 1 and 2) ≈ Within ℜ*_g_* (%) (CNN- Choices 1 and 2). Moreover, it can be noticed that within ℜ*_g_* (%) (MLP—Choices 3 and 4) > within ℜ*_g_* (%) (CNN—Choices 3 and 4). In other words, the percentage of weights within ℜ*_g_* (%) for MLP and Choices 3 and 4 is greater by approximately 0.25%.

Since we have almost the same accuracies: Accuracy (%) (MLP—Choice 3) ≈ Accuracy (%) (MLP—Choice 4) and Accuracy (%) (CNN—Choice 3) ≈ Accuracy (%) (CNN—Choices 4), one can observe almost the same difference in accuracy of 0.9% in favor of CNN for Choices 3 and 4, which has been intuitively expected. In addition, it is also reasonable to analyze the results shown in Figure 12a for a somewhat narrower range of ℜ*_g_*. More precisely, if we analyze the results up to *w*_max_ = 6.469376 (Choice 1 within ℜ*_g_* (%) is 99.9999% of the weights), a somewhat smaller value representing the accuracy dynamics can be determined. From both analyses conducted for the CNN model, trained on the MNIST dataset, and quantized by using three-bit UQ, one can conclude that a fairly stable accuracy can be achieved regardless of the ℜ*_g_* choice, which is not the case with the post-training two-bit uniform quantization reported for the MLP case in [34].

In this paper, results are also provided for both specified NN models, MLP and CNN, trained on the Fashion-MNIST dataset [44]. Table 5 and Table 6 present SQNR and the QNN model accuracy obtained with the application of different three-bit UQ designs for MLP and CNN trained on the Fashion-MNIST dataset, where ℜ*_g_* is defined following the same principles as in previous tables.

Let us first highlight again that the MLP model achieves an accuracy of 98.1% on the MNIST validation set and 88.96% on the Fashion-MNIST validation set, where weights are represented in the FP32 format. Compared to MLP, the CNN model achieves a higher accuracy of 98.89% on the MNIST validation set, as well as a higher accuracy of 91.53% obtained on the Fashion-MNIST validation set, with weights also represented in the FP32 format. The highest obtained QNN accuracy for the observed ℜ*_g_* ranges in Table 5 amounts to 88.48% for Choice 4, while the lowest value for Choice 2 is 87.12%. Moreover, the accuracy dynamics for the results presented in Table 5 amounts to 1.36%. For Choice 4, we can calculate that the degradation of the accuracy due to the application of the three-bit UQ amounts to 88.96% −88.48% = 0.48%. In Table 6, the highest obtained accuracy amounts to 88.02% for Choice 3 (degradation due to the applied three-bit UQ amounts to 91.53% − 88.02% = 3. 52%), while the lowest value, also for Choice 2, is 84.90%. The accuracy dynamics for the results presented in Table 6 amounts to 4.12%. It has been highlighted in [47] that Choice 2 is a popular choice for the quantizer design, which, as one can notice from Table 5 and Table 6, is not the most suitable choice in terms of accuracy and SQNR, especially when the amplitude dynamics of the weights being quantized is relatively large.

**Figure 13:**
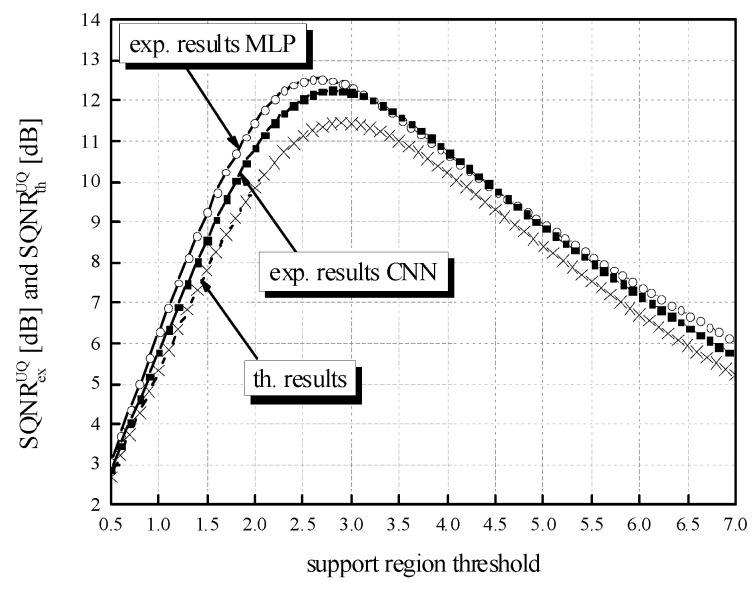
Theoretical and experimental SQNR values for MLP and CNN (both trained on Fashion-MNIST dataset) and for a wide range of *w*_supp_ values assumed in three-bit post-training UQ.

In order to inspect further the influence of the ℜ*_g_* choice on both, the accuracy and SQNR, for a wide range of *w*_supp_ values and for both NN models (MLP and CNN) trained on Fashion-MNIST dataset, we can analyze results presented in Figure 12b,c. For *w*_supp_ having a relatively wide range of values, the accuracy dynamics presented in Figure 12b,c amount to 2.5% and 5.5%, for MLP and CNN, respectively. This indicates that in the case where the specified MLP and CNN are trained on Fashion-MNIST dataset, more careful choice of ℜ*_g_* should be performed. In addition, results presented in Figure 12b,c indicate that there is a number of choices for ℜ*_g_* that can result in the degradation of accuracy due to the application of the three-bit UQ up to 1% compared to the case with the highest identified accuracy. Eventually, we can highlight that the highest accuracies for the observed Fashion-MNIST dataset and MLP and CNN models are achieved for *w*_supp_ = 3.42 and *w*_supp_ = 3.52, respectively. These highest accuracies amount to 88.78% and 89.54% for MLP and CNN models (for Fashion-MNIST dataset), respectively. In other words, with the observed three-bit UQ, and with the selection of *w*_supp_ = 3.42 and *w*_supp_ = 3.52 for the corresponding cases (MLP and CNN), the accuracy degradation of 88.96% − 88.78% = 0.18% and 91.53% − 89.54% ≈ 2% is achieved. We can derive additional conclusions by analyzing theoretical and experimental SQNR values for MLP and CNN (both trained on Fashion-MNIST dataset) determined for a wide range of *w*_supp_ values assumed in three-bit post-training uniform quantization (see Figure 13).

**Figure 14:**
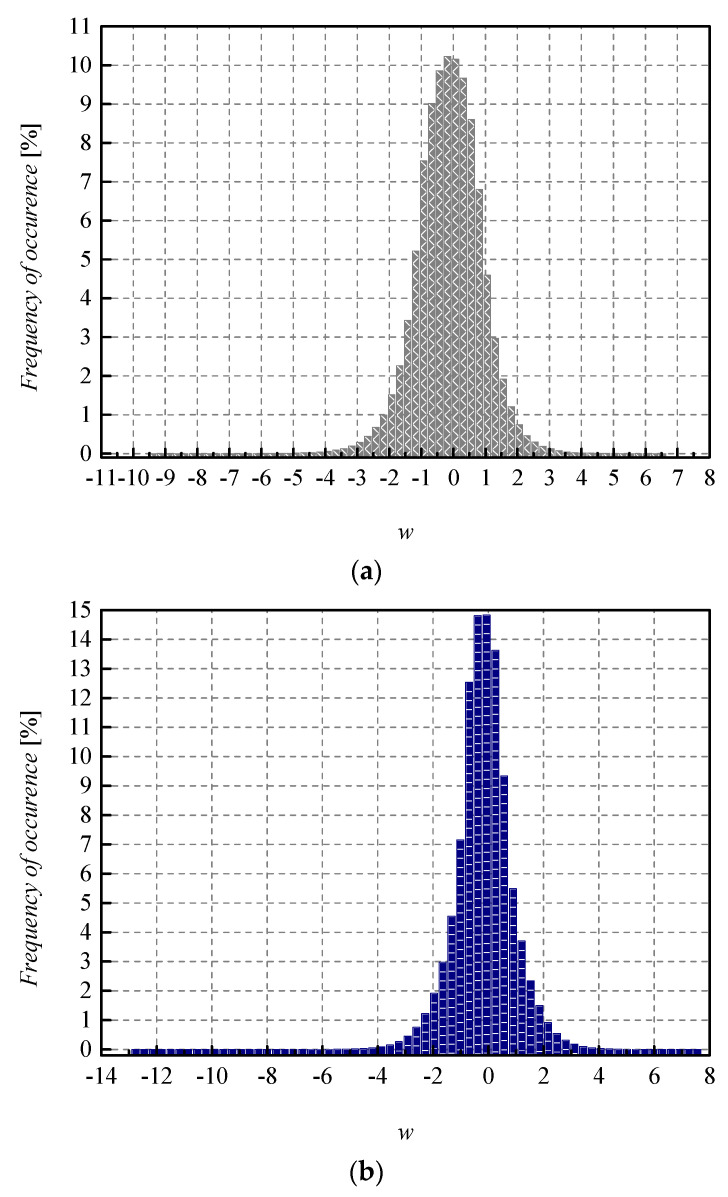
Normalized histogram of weights (FP32) for (**a**) MLP and (**b**) CNN trained on Fashion-MNIST.

From the SQNR aspect, the results presented in Table 5 and Table 6 show that, as expected, the highest values in both cases of interest (MLP and CNN) are achieved for Choice 4. As already highlighted, the theoretical SQNR curve decreases after passing the theoretically optimal value of 2.9236. Additionally, as previously concluded for the MLP and CNN trained on the MNIST dataset, in the observed cases with MLP and CNN trained on the Fashion-MNIST dataset, this theoretically optimal value of *w*_supp_ differs from *w*_supp_ = 2.7 and *w*_supp_ = 2.8, at which the maximum of the experimentally determined SQNR value of 12.5374 dB and 12.2462 dB are achieved for MLP and CNN case, respectively. Similarly, as we have explained for the MNIST dataset and MLP and CNN, we can here explain that these differences are the consequence of lower amplitude dynamics of real normalized weights (see Figure 14) used in the experimental analysis compared to the theoretically assumed ones. For *w*_supp_ = 2.7, the QNN model’s accuracy for MLP trained on the Fashion-MNIST dataset amounts to 88.54%, which is not the maximum obtained accuracy in the experiment. A similar conclusion can be derived for the CNN trained on the Fashion-MNIST dataset, where for *w*_supp_ = 2.8, we have determined that the QNN model’s accuracy is 87.73%. Therefore, we can conclude that for both NN models (MLP and CNN) and for both datasets (MNIST and Fashion-MNIST), it is not of utmost importance to determine the optimal value of the support region threshold, as it is the case in classical quantization, in order to fit into some predefined range of accuracy degradation that can be considered acceptable.

As with the MNIST dataset, in the case of using the Fashion-MNIST dataset, one can notice the higher amplitude dynamics of weights for the CNN case compared to the MLP case (see Figure 14). For that reason, the maximum of experimentally obtained SQNR values in the CNN case is shifted right in Figure 13. Moreover, one can notice that the theoretically determined SQNR curve is below both experimentally determined SQNR curves, where the reasons for these differences are similar to those explained for the MNIST dataset.

In summary, the experimental analysis confirmed that for the case of *R* = 3 bit/sample, different choices of ℜ*_g_* do not have an equally high impact on the QNN model’s accuracy as is the case when the bit rate is equal to 2 bits per sample. Moreover, it has been shown that if the support region threshold takes value from a properly defined wide range, for both specified NN models, MLP and CNN trained on MNIST dataset, the performance of QNNs is quite stable for various choices of ℜ*_g_*, and the accuracy of QNNs are greatly preserved in comparison to the corresponding baseline NN models. Eventually, we have concluded that for both NN models (MLP and CNN) and for both datasets (MNIST and Fashion-MNIST), it is not of utmost importance to determine the optimal value of the support region threshold, as it is the case in classical quantization, in order to fit into some predefined range of accuracy degradation that can be considered acceptable.

## 7. Summary and Conclusions

In this paper, we have shown that when three-bit UQ is utilized for post-training quantization, the accuracies of two NNs (MLP and CNN) that we have pretrained for the MNIST dataset can be preserved for various choices of the key parameter of the quantizer in question. The degradation of accuracies has been estimated relative to the ones of the corresponding baseline NNs with weights represented in FP32 format, which is the default format used on platforms that utilize GPUs and CPUs. We have also shown that in post-training three-bit uniform quantization, for both NN models (MLP and CNN) and for two datasets (MNIST and Fashion-MNIST), it is not of utmost importance, as it is in classical quantization, to determine the optimal support region threshold value of the UQ to achieve some predefined accuracy of the QNN. Opposite to the case with two-bit uniform quantization, where we intuitively anticipated and showed that the choice of the support region width has a high impact on the accuracy of the QNN model, in this paper, for the same classification task and the same specified MLP, we have not given such anticipation in the case of the UQ having only one additional bit. We have actually examined whether the choice of the support region width of the three-bit UQ has an impact on the accuracy of the QNN, and we have determined weak dependence of the accuracy on the support region threshold, especially for the MLP model. Therefore, we have highlighted that an unsuitable choice of the support region of the two-bit UQ can significantly degrade the accuracy of QNN, whereas tunning the support region threshold value is far simpler and less stringent in the case with three-bit UQ. We have shown that by using three-bit UQ to compress weights of MLP and CNN (trained on MNIST dataset) more than 10 times, we have managed to significantly preserve the accuracy of the NN model, which is degraded by 0.13% and 0.10%, respectively. The simplicity of our proposal, along with the high robustness of accuracy in changing the support region threshold value, indicates that the post-training quantization model addressed in this paper can be very exploited in a simple way, which is especially important in edge devices for real-time classification tasks. In other words, since the addressed low-bit quantization can be considered as an attractive and powerful compression technique that could enable QNN to fit in the edge device with accuracy preserved to a high extent, one can expect that the analysis performed in the paper will also have great practical importance. Eventually, for future work, we plan to experiment with some other datasets and with very simple quantizer models, as the one utilized in this paper.

## Figures and Tables

**Table 1:** Analysis of the MLP weight’s statistics: amplitude dynamics before and after the normalization to zero mean and unit variance, the accuracy before and after three-bit UQ quantization (FP32 and UQ) and SQNR.

Weights File Name	min Weights FP32	max Weights FP32	min Weights Norm FP32	max Weights Norm FP32	acc. FP32 (%)	acc. UQ[H] (%)	SQNR UQ[H] (dB)
mnist_weights_used	−0.5185	0.3447	−7.0638	4.8371	98.1	97.66	12.9455
mnist_weights_1	−0.4948	0.3594	−6.6845	5.0123	98.28	98.17	12.9623
mnist_weights_2	−0.5122	0.3533	−6.960	4.948	98.18	97.9	12.9021
mnist_weights_3	−0.5096	0.3624	−6.9375	5.0808	98.3	97.92	12.9204

**Table 2:** SQNR and QNN accuracy for MLP trained on the MNIST dataset with the application of different three-bit UQ designs.

*w*_min_ = −7.063787 *w*_max_ = 4.8371024 *x*_max_[H] = 2.9408 *x*_max_[J] = 2.9236	Choice 1 ℜ*_g_* [−*w*_max_, *w*_max_]	Choice 2 ℜ*_g_* [*w*_min_, −*w*_min_]	Choice 3 ℜ*_g_* [−*x*_max_[H], *x*_max_[H]]	Choice 4 ℜ*_g_* [−*x*_max_[J], *x*_max_[J]]
SQNR_ex_^UQ^(dB)	9.2302	5.9005	12.9455	12.9766
SQNR_th_^UQ^(dB)	8.6901	5.1273	11.4414	11.4419
Accuracy (%)	97.85	97.85	97.66	97.65
Within ℜ*_g_* (%)	99.988	100	99.403	99.381

**Table 3:** SQNR and QNN accuracy for MLP trained on MNIST dataset with the application of different two-bit UQ designs [34].

*w*_min_ = −7.063787 *w*_max_ = 4.8371024 *x*_max_[H] = 1.9605 *x*_max_[J] = 2.1748	Case 1 ℜ*_g_* [−*w*_max_, *w*_max_]	Case 2 ℜ*_g_* [*w*_min_, −*w*_min_]	Case 3 ℜ*_g_* [−*x*_max_[H], *x*_max_[H]]	Case 4 ℜ*_g_* [−*x*_max_[J], *x*_max_[J]]
SQNR_ex_^UQ^(dB)	2.8821	−1.2402	8.7676	8.7639
SQNR_th_^UQ^(dB)	1.9360	−2.0066	6.9787	7.0707
Accuracy (%)	96.97	94.58	96.34	96.74
Within ℜ*_g_* (%)	99.988	100	94.787	96.691

**Table 4:** SQNR and accuracy—the application of different three-bit UQ designs for CNN and MNIST dataset.

*w*_min_ = −8.372064 *w*_max_ = 6.469376 *x*_max_[H] = 2.9408 *x*_max_[J] = 2.9236	Choice 1 ℜ*_g_* [−*w*_max_, *w*_max_]	Choice 2 ℜ*_g_* [*w*_min_, −*w*_min_]	Choice 3 ℜ*_g_* [−*x*_max_[H], *x*_max_[H]]	Choice 4 ℜ*_g_* [−*x*_max_[J], *x*_max_[J]]
SQNR_ex_^UQ^(dB)	6.5399	4.0532	12.8594	12.8812
SQNR_th_^UQ^(dB)	5.9846	3.4347	11.4414	11.4419
Accuracy (%)	98.38	97.64	98.56	98.56
Within ℜ*_g_* (%)	99.9999	100	99.165	99.136

**Table 5:** SQNR and accuracy—the application of different three-bit UQ designs for MLP and Fashion-MNIST dataset.

*w*_min_ = −9.395458 *w*_max_ = 6.533294 *x*_max_[H] = 2.9408 *x*_max_[J] = 2.9236	Choice 1 ℜ*_g_* [−*w*_max_, *w*_max_]	Choice 2 ℜ*_g_* [*w*_min_, −*w*_min_]	Choice 3 ℜ*_g_* [−*x*_max_[H], *x*_max_[H]]	Choice 4 ℜ*_g_* [−*x*_max_[J], *x*_max_[J]]
SQNR_ex_^UQ^(dB)	6.665	3.1292	12.3843	12.40141
SQNR_th_^UQ^(dB)	5.8894	2.2619	11.4414	11.4419
Accuracy (%)	87.69	87.12	88.39	88.48
Within ℜ*_g_* (%)	99.997	100	99.027	98.999

**Table 6:** SQNR and accuracy—the application of different three-bit UQ designs for CNN and Fashion-MNIST dataset.

*w*_min_ = −12.76407909 *w*_max_ = 7.5561204 *x*_max_[H] = 2.9408 *x*_max_[J] = 2.9236	Choice 1 ℜ*_g_* [−*w*_max_, *w*_max_]	Choice 2 ℜ*_g_* [*w*_min_, −*w*_min_]	Choice 3 ℜ*_g_* [−*x*_max_[H], *x*_max_[H]]	Choice 4 ℜ*_g_* [−*x*_max_[J], *x*_max_[J]]
SQNR_ex_^UQ^(dB)	4.9069	−0.579776	12.2284	12.2341
SQNR_th_^UQ^(dB)	4.4615	−0.9315	11.4414	11.4419
Accuracy (%)	85.53	84.901	88.02	87.97
Within ℜ*_g_* (%)	99.998	100	98.822	98.784

## Data Availability

The data used to support the findings of this study are available at http://yann.lecun.com/exdb/mnist/ and at https://github.com/zalandoresearch/fashion-mnist (accessed on 10 December 2021).
